# Efficacy of disinfection procedures to reduce *Acinetobacter baumanii bla*_OXA-23_ contamination rate of needleless connectors: an in-vitro study

**DOI:** 10.1016/j.infpip.2023.100328

**Published:** 2023-12-01

**Authors:** C. Biazus-Dalcin, T.C.M. Sincero, C.P. Zamparette, D.C. Tartari, S. de Souza, T.L. Silva, A. Tomazoni, P.K. Rocha

**Affiliations:** a School of Health Sciences, University of Dundee, Dundee, UK; b Department of Clinical Analyses, Health Sciences Centre, Universidade Federal de Santa Catarina (UFSC), Florianópolis, Brazil; c The State of Santa Catarina, Florianópolis, Brazil; d Nursing Department, Health Sciences Centre, Universidade Federal de Santa Catarina (UFSC), Florianópolis, Brazil; e Hospital de Clínicas of the Universidade Federal do Paraná (UFPR), Curitiba, Brazil

**Keywords:** Acinetobacter, Bacterial load, Catheters, Catheter-related infections, Disinfection

## Abstract

**Aim:**

This study aimed to verify the efficacy of disinfection procedures to reduce *Acinetobacter baumannii bla*_OXA-23_ bacterial load in needleless connectors that had been experimentally contaminated.

**Methods:**

Two-way intermediate extender's hub and needle-free valve were contaminated with *Acinetobacter baumannii bla*_OXA-23_. To disinfect them, the following procedures were carried out: sterile gauze with 70% ethanol, sterile gauze with Incidin®, and 70% isopropyl alcohol single-use cap, with eight times friction for 10 s, followed by 5 s drying time. The statistical tests Kruskal–Wallis and post-hoc Conover were performed using MedCalc®.

**Results:**

A total of 82 experiments were conducted. All tested disinfection procedures were efficacious in reducing the A. *baumannii bla*_OXA-23_ load. The 70% IPA single-use cap was found to be the best method for disinfecting the two-way intermediate extender's hub (87.28%), while all the methods were efficacious for the disinfection of the needle-free valve (more than 90%). During the inoculation period, *A. baumannii bla*_OXA-23_ showed less adherence to the needle-free valve during the inoculation period, probably due to the device's design.

**Conclusion:**

The three tested disinfection procedures using sterile gauze with 70% ethanol, sterile gauze with Incidin®, and 70% IPA single-use cap were found to be efficacious in reducing the bacterial load of *A. baumanni bla*_*OXA-23*_ in needleless connectors. Proper disinfection of needleless connectors is a crucial nursing practice to prevent bloodstream infections, as it significantly reduces the bacterial load present in the device.

## Introduction

*Acinetobacter baumannii* is the is the fourth most commonly occurring micro-organism in bloodstream infections (BSIs) associated with central venous catheter (CVC) in Brazil [1]. Infections caused by multi-resistant *A. baumannii* that produce oxacillinase-type carbapenemases (OXA) are currently considered a clinical and epidemiological emergency. In some wards, the presence of *A. baumannii* producing OXA-23 has acquired an endemic character, highlighting the need for studies focusing on the prevention of infections caused by this pathogen, as well as policies to control its spread [2,3].

In the context of BSIs caused by *A. baumannii bla*_OXA-23_, infection prevention is crucial for ensuring patient safety. One of the important actions that healthcare professionals must consider is the disinfection of needleless connectors (NCs) as they are attached to catheter hubs. Disinfecting NCs involves using physical or chemical methods to minimize the number of pathogenic micro-organisms present on the surface [4].

A study conducted in São Paulo, Brazil, suggests that disinfecting NCs before and after handling them, along with other procedures to prevent contamination, is crucial to minimizing the contamination rate at the surface of NCs [5]. Another study found that bacterial contamination was present in 44% of the NCs in the intensive care unit (ICU) [6].

The guidelines on NC disinfection vary. The Centres for Disease Control and Prevention (CDC) [7], National Health Surveillance Agency (Anvisa, Brazil) [8], Infusion Nursing Society (INS) [9], Epic3: National Evidence-Based Guidelines for Preventing healthcare-associated infections [4] and Royal College of Nursing (RCN) [10] do not specify the optimal disinfection procedure for the surface of NCs. The evidence for successful NC disinfection ranges from a minimum of 5 s of scrub time, and there is not always a clear specification of the drying time that should be allowed or the type of disinfectant product that should be used (such as chlorhexidine >0.5 in 70% isopropyl alcohol (IPA) or 70% IPA).

Disinfecting devices such as NCs used in peripheral inserted CVCs (PIVCs) and CVCs is crucial for safe nursing practice. However, most guidelines regarding NC disinfection practices are based on the standards followed in the wealthiest countries such as the USA, Australia and European countries [7,9,11,12]. It is important for other countries to conduct initial studies that reflect their respective clinical practices, especially in Latin American countries and other medium- or low-income countries. The Brazilian reality could be taken as an example for such studies.

The aim of this study was to establish the efficacy of disinfection procedures in reducing the bacterial load of *A. baumannii bla*_OXA-23_ in NCs.

## Methods

An in-vitro experimental study tested three disinfection procedures on two different NCs artificially contaminated with a strain of *A. baumannii bla*_OXA-23_ LA216 [13].

The NCs used in this study were the two-way intermediate extender's hub (Polifix®, B. Braun), and the needle-free valve (Safeflow®, B. Braun).

The disinfectant procedures included were as follows: (1) take a sterile gauze and apply 1 mL of 70% ethanol, use mechanical friction to rotate the gauze in clockwise and counterclockwise direction for 10 s (180°), allow the area to dry for 5 s; (2) take a sterile gauze and apply 1 mL of Incidin® (Dräger), use mechanical friction to rotate the gauze in clockwise and counterclockwise directions for 10 s (180°), allow the area to dry for 5 s; and (3) rotate the 70% IPA single-use cap (Site-Scrub®, Becton Dickinson, BD) eight times clockwise and counterclockwise (for a total of 10 s) (180°), followed by 5 s of drying time as per the manufacturer's instructions.

It is important to note that all procedures were conducted under sterile conditions using sterile materials. Each disinfection experiment was conducted in triplicate for technical replicates, and on different days. Three biological replicates were performed to ensure the reliability and reproducibility of the proposed method. Non-treated controls (NTCs) and sterility controls (negative controls) were also performed in all experiments. The NTCs were performed with bacterial contamination, without using any treatment.

### Bacterial inoculum and contamination of NCs

A well-characterized strain of *A. baumannii bla*_OXA-23_ [13] was cultured and prepared as per the recommended guidelines, *Methods for Dilution Antimicrobial Susceptibility Tests for Bacteria That Grow Aerobically* [14]. The initial bacterial suspension (iBS), which contains ∼1.5 × 10^8^ CFU (colony forming units), was prepared by adjusting the turbidity of McFarland Scale 0.5 (Newprov®) and then measuring it electronically using the DensiCHEK™ turbidimeter (BioMerieux®).

To contaminate the NCs, iBS was diluted 100 times by mixing 10 μL of BS-A with 990 μL of 0.9% sterile saline solution. The resulting work bacterial suspension (wBS) contained 1.5 × 10^6^ cfu. The NC devices were then immersed in 1 mL of the wBS in 15-mL sterile conical centrifuge graduated tubes for a timed duration of 5 min. After contamination, the devices were removed from the conical tubes using forceps and left to dry in a closed Petri dish for 60 min at 35 ± 1°C in a bacteriological oven SL-101 (SOLAB).

### Disinfection procedures

The devices, except for the non-treated controls, underwent three different disinfection procedures after being contaminated. These procedures were carried out according to the manufacturer's instructions or in line with the recommended guidelines of clinical practice, such as those of INS [9], CDC [7] and Anvisa (Brazil) [8].

All disinfection procedures were conducted by the researcher using the same dominant hand. Table 1 summarizes the disinfection procedures.

**Table 1:** Disinfection procedures used in the study

Device	Device surface	Disinfectant product	Friction	Friction time	Drying time
Two-way intermediate extender's hub	External with cap	Sterile gauze with 1 mL 70% ethanol	Rotate the gauze in clockwise and counterclockwise directions (180°)	10 s	5 s
	External with cap	Sterile gauze with 1 mL Incidin® (Dräger)	Rotate the gauze in clockwise and counterclockwise directions (180°)	10 s	5 s
	Internal without cap	70% IPA single-use cap (Site-Scrub®, BD)	Rotate the device in clockwise and counterclockwise directions eight times (180°)	10 s	5 s
Needle-free valve	External	Sterile gauze with 1 mL 70% ethanol	Rotate the gauze in clockwise and counterclockwise directions (180°)	10 s	5 s
	External	Sterile gauze with 1 mL Incidin® (Dräger)	Rotate the gauze in clockwise and counterclockwise directions (180°)	10 s	5 s
	External	70% IPA single-use cap (Site-Scrub®, BD)	Rotate the device in clockwise and counterclockwise directions eight times (180°)	10 s	5 s

IPA, isopropyl alcohol.

When disinfecting with 70% ethanol or Incidin®, 1 mL of the respective disinfectant was pipetted over the gauze for both the needle-free valve and the two-way intermediate extender's hub (with cap) situated outside the NC. Using forceps, the NC device was held, and the gauze was rotated clockwise and counterclockwise for 10 s (180°) with mechanical friction. The devices were then left to dry for 5 s after disinfection.

The 70% IPA single-use cap (Site-Scrub®) disinfection procedures were performed in accordance with the manufacturer's recommendations. Different approaches were used in the two NCs. For the needle-free valve, the instructions were to remove the seal of Site-Scrub®, hold the needle-free valve with forceps, and apply mechanical friction by placing the Site-Scrub® in the middle of the needle-free valve; then, rotate the Site-Scrub® clockwise and counterclockwise eight times for 10 s (180°). Similarly, for the two-way intermediate extender's hub, the seal of Site-Scrub® was removed, and the cap was removed from the two-way intermediate extender's hub. Then, holding the two-way intermediate extender hub device with forceps, mechanical friction was applied by rotating the Site-Scrub® clockwise and counterclockwise eight times for 10 s (180°) in the centre of the connector. After disinfection, both the NC needle-free valve and the two-way intermediate extender's hub were dried for 5 s.

### Bacterial quantification

All of the treated NCs and controls were immersed in a 15-mL conical centrifuge tube filled with 2 mL of 0.9% saline solution. The tubes were sealed, and vigorously mixed at maximum speed for 5 min using a Phoenix MOD AT56 vortex mixer. Subsequently, they were placed in an ultrasonic bath (UltraCleaner 1400 Unique) operating at a frequency of 40 kHz for 10 min.

After vortex homogenization, 100 μL of each sonicated suspension was evenly spread over tryptic soy agar (TSA) in Petri dishes using a sterile disposable Drigalski T-shaped handle. The handle was moved in a circular motion until the suspension was dried. The Petri dishes were then incubated at 35 ± 1°C for 24 h in a bacteriological incubator (SL-101 from SOLAB) before counting cfu.

### Statistical analyses

The study compared the number of CFU recovered from treated NCs and NTCs. The database was created using Microsoft Excel® Software 2016 (USA), and statistical analyses were conducted using MedCalc® software, version 19.1.7 (Belgium). The Kruskal–Wallis non-parametric test was utilized to examine and compare independent groups, and the Conover test was used for post-hoc analysis to determine the interaction between the disinfection procedures. Statistically significant differences were considered when the *P*-value was less than 0.05.

## Results

Of the 90 experiments conducted, 82 were considered for the findings as eight technical outliers were excluded. All the negative controls displayed the sterilization of the materials used in the research. Thirty-seven experiments were performed using two-way intermediate extender's hub, while the needle-free valve was used in 45 experiments.

During the study, it was observed that the NC two-way intermediate extender's hub (used in PIVC) and the needle-free valve (used in PIVC and CVC) showed different results when it came to the adherence of *A. baumannii bla*_*OXA-23*_. The NC two-way intermediate extender's hub had a higher number of bacterial cells adhered, compared with the needle-free valve. The NTC in the two-way intermediate extender's hub presented 342 CFU, while the needle-free valve showed 110 CFU, which shows that 67.83% less bacterial cells adhered. This can be explained by the fact that the needle-free valve has fewer grooves as compared with the NC's design.

The experiments conducted in the two-way intermediate extender's hub showed that all disinfection procedures were efficacious. Among them, the 70% IPA single-use cap was the most efficacious, reducing the bacterial load by 87.28%, while the least efficacious was sterile gauze with 70% ethanol, with only 48.54% efficacy (Supplementary Table S1).

There was no difference in efficacy between sterile gauze treated with 70% ethanol and sterile gauze treated with Incidin®, with 48.53% and 48.88%, respectively, of bacterial load reduction.

**Figure 1:**
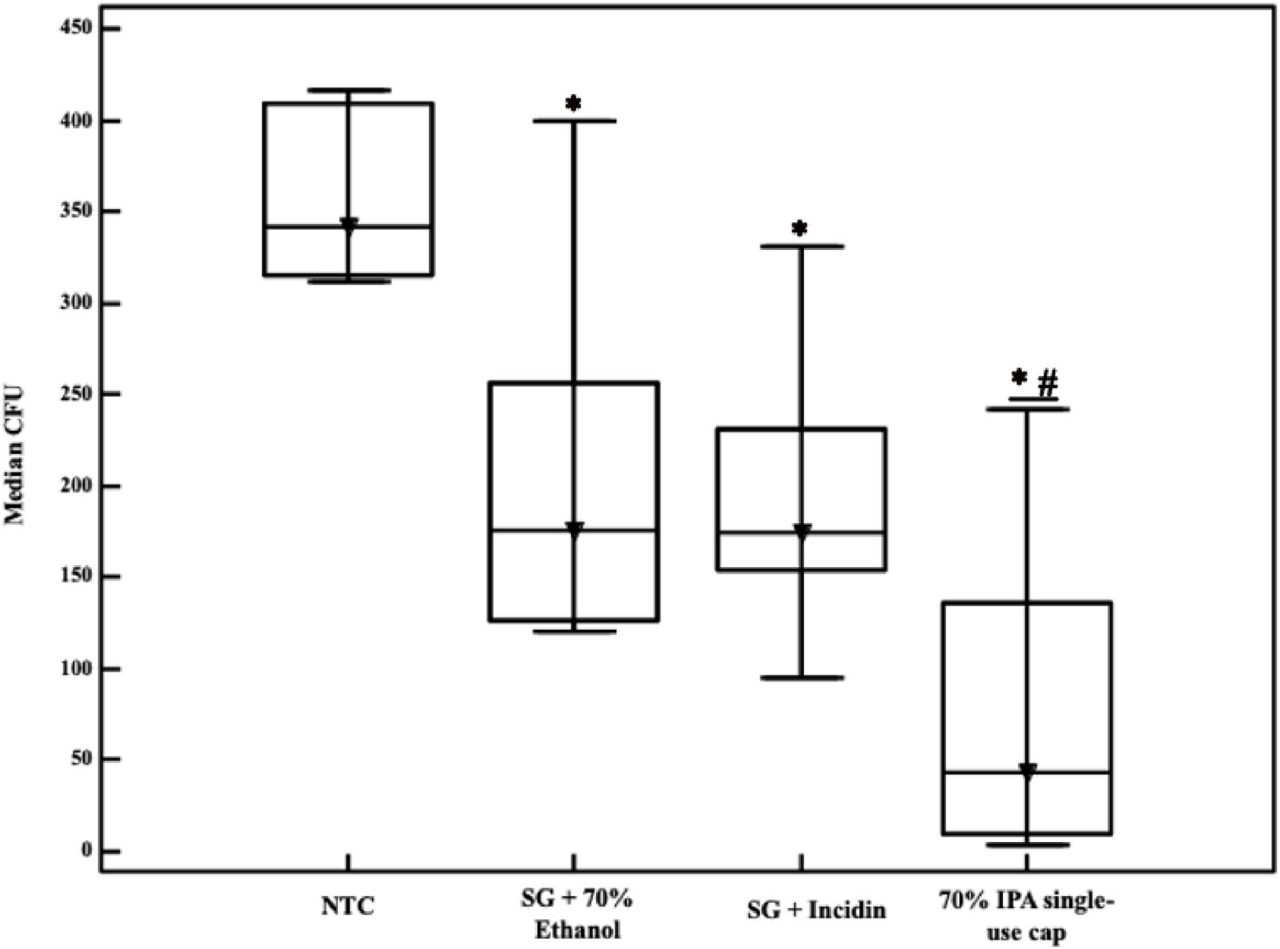
Microbial burden after disinfection on two-way intermediate extender's hub, between control and treatment groups. CFU, colony forming units; IPA, isopropyl alcohol; NTC, non-treated control; SG, sterile gauze. ∗ Different from NTC. # Different from other treatments.

The experiment showed that the disinfection procedures used in two-way intermediate extender's hub were efficacious with a significance level of *P*=0.000812. Figure 1 compares the reduction in CFU in the interquartile range (sample variation) and median among all disinfection procedures. The 70% IPA single-use cap was the most efficacious disinfection procedure for the two-way intermediate extender's hub.

Related to the experiments conducted in the needle-free valve, it was observed that all disinfection procedures were efficacious. The three disinfection procedures exhibited varying degrees of reduction ranging from 94.54% (70% IPA single-use cap) to 97.27% (sterile gauze with 70% ethanol) (Supplementary Table S2).

**Figure 2:**
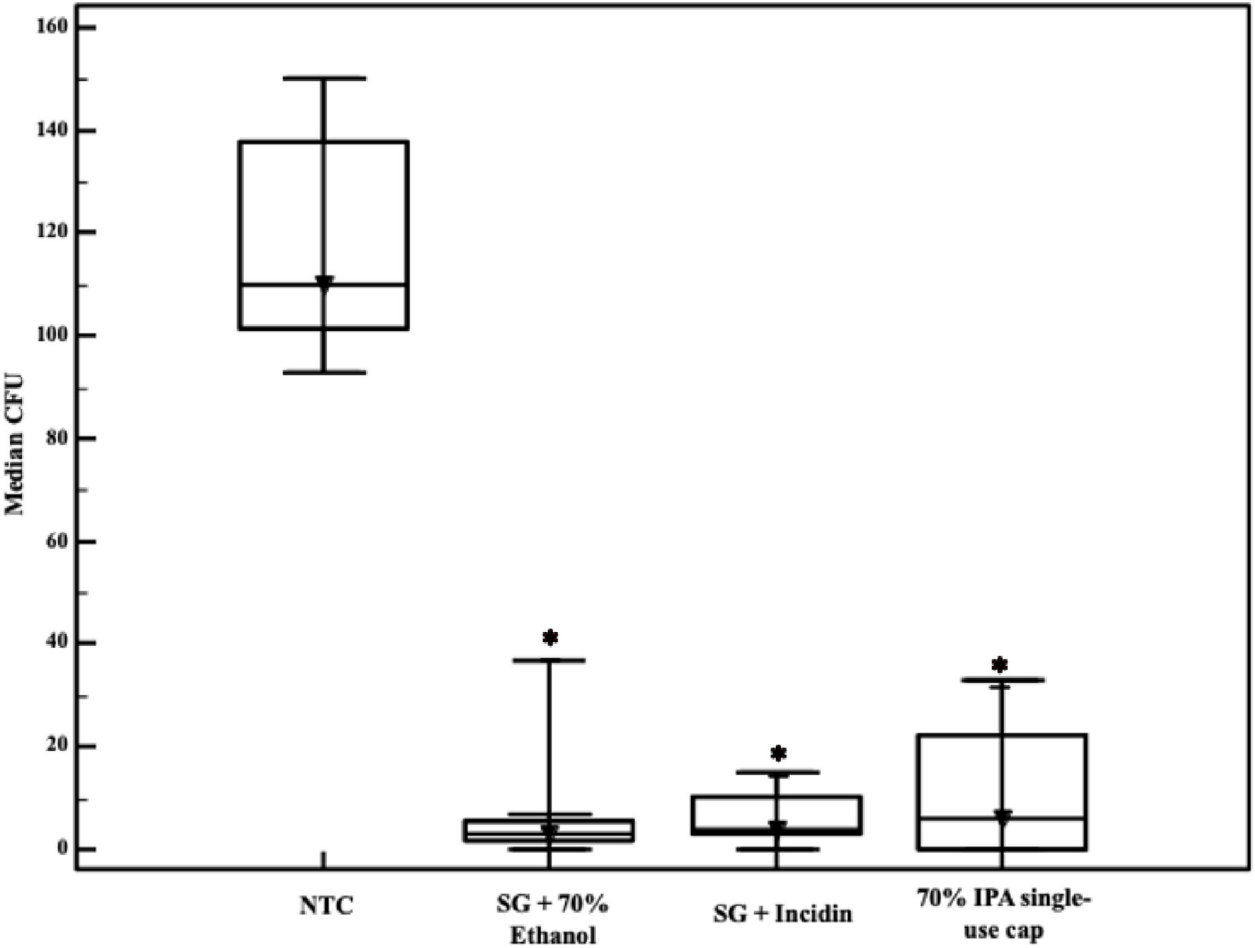
Microbial burden after disinfection on needle-free valve in peripheral venous catheters, between control and treatment groups. CFU, colony forming units; IPA, isopropyl alcohol; NTC, non-treated control; SG, sterile gauze. ∗ Different from NTC.

Based on the samples presented, it can be observed that the variance in the methods used was noticeable. The experiment conducted with sterile gauze and 70% ethanol showed a higher variance than the other methods. However, all methods of disinfection were efficacious, and there were no significant differences between the different treatments, as shown in Figure 2.

## Discussion

In this in-vitro study, all the tested disinfection procedures were efficacious in reducing the *A. baumannii bla*_OXA-23_ load in NCs in a controlled environment. This is important because *A*. *baumannii bla*_OXA-23_ is an opportunistic pathogen, that can survive for extended periods in hospital settings, especially on inanimate surfaces – such as the catheter's connectors [15].

Inadequate NC decontamination can result in device contamination, which can lead to BSI [7]. According to the RCN [10], the cleaning, disinfection and sterilization processes for NCs should follow local policy and comply with manufacturers' guidelines. The protocols for these processes should be clearly stated in the organizational policies and procedures. However, the current guidelines vary in recommendations for disinfection procedures, making it challenging to understand and follow the best practices for NC disinfection [[7], [8], [9]].

In Brazil, Anvisa recommends the use of alcohol-based antiseptic solutions for disinfection, but it does not specify examples of disinfectant products to use or how much to use [8]. The guidelines encourage scrubbing the hub for 5–15 s. This study followed Anvisa [8] and other international guidelines by using a 10-s friction time for all the procedures [4,9,10]. A previous systematic review [16] showed that the greatest risk for catheter contamination after insertion is the contamination of the NC, with 33–45% of NCs being contaminated. Moreover, it showed compliance with disinfection as low as 10%. The review recommended scrubbing with 70% alcohol for 5–60 s and disinfecting NCs before any connection. Another systematic review with meta-analysis [17] showed that using alcohol-impregnated caps and alcoholic chlorhexidine gluconate together is more effective in reducing the rates of central line associated blood stream infection (CLABSI) than using 70% alcohol wipes.

In this experimental study, it was found that 70% IPA was the most efficacious disinfection procedure for the two-way intermediate extender's hub. However, it should be noted that the experiment was conducted without the external cap that usually covers the hub, which was not in accordance with the manufacturer's recommendations. This could potentially affect the results and should be taken into consideration when analysing the findings.

A clinical trial conducted in the USA and registered in the National Library of Medicine showed that 70% IPA disinfection solution was effective in achieving zero colony formation in NCs across the 295 units analysed [18]. The study found that there was no significant difference between the effectiveness of disinfection achieved through a 5-s scrub or a 15-s scrub with IPA.

All of the disinfection procedures were efficacious in reducing contamination of the needle-free valve, with rates of above 90% reduction. However, previous studies did not include the use of Incidin® as a chemical disinfectant because it is not a standard method in international guidelines for NC disinfection [7,9]. The research team acknowledges that other active measures, such as bundles, are necessary to control catheter-related infections [19,20].

In the study, it was found that the needle-free valve had 67.83% less bacterial load adherence than the two-way intermediate extender's hub when used as an NTC. The adherence of *A. baumannii* to the needle-free valve was also found to be less. This finding was not premeditated. The literature shows that depending on NC design, it can retain more microbial cells [21]. With a vast number of NCs available, it is important that nurses know which to use and how to disinfect them effectively. Future randomized controlled trials focusing on the practice of disinfection procedures in Brazil are a necessary step in the prevention of catheter-related BSI.

It is essential to develop safer practices in Brazil to reduce infection rates. International studies typically focus on the use of individual sterile pads with pre-applied disinfectant solution, called wipes/swabs [9,7,11,12,22]. However, some Brazilian hospitals use gauze with disinfectant products for NC disinfection procedures.

It should be noted that this study has some limitations. Firstly, the pressure used in each disinfection procedure was not measured, even though all disinfection procedures were performed with the dominant hand of a single researcher. It is important to consider that different pressures can lead to different outcomes, and further research is required to analyse this aspect [23]. Secondly, future studies should test different disinfection times to determine the reduction of bacteria. Additionally, it is important to highlight that the efficacy of the Site-Scrub® device was tested only with 70% IPA, and this may have affected the results. Therefore, the efficacy can be associated with both the disinfectant product used (70% IPA) and the Site-Scrub® device. Lastly, this study only focused on the *A*. *baumannii bla*_*OXA-23*_ strain and did not consider other prevalent micro-organisms, such as coagulase-negative Staphylococcus [5,24]. This decision was made due to the availability of the strains and the timeframe of the study.

In conclusion, the study found that all three disinfection procedures were efficacious in reducing the bacterial load of *A. baumannii bla*_*OXA-23*_. Among the three procedures tested, the most efficacious one in disinfecting the two-way intermediate extender's hub was the 70% IPA single-use cap, rotated clockwise and counterclockwise eight times (180°) for 10 s, followed by 5 s of drying time.

This study also found that there was lower adherence of *A. baumanni bla*_*OXA-23*_ in the needle-free valve compared with the two-way intermediate extender's hub. This reduction amounted to almost 68%. One of the possible reasons is the needle-free valve design, which has fewer grooves. Based on this, it is recommended to use needle-free valves in nursing practice.

It is important that future studies be conducted to consider procedure variations, such as the pressure during the disinfection procedure and the amount of disinfectant products used.

## CRediT author statement

**Camila Biazus Dalcin:** Conceptualization, Methodology, Investigation, Formal analysis, Writing - Original Draft, Review & Editing.

**Thaís Cristine Marques Sincero:** Methodology, Formal analysis, Writing - Review & Editing.

**Caetana Paes Zamparette:** Methodology, Formal analysis, Writing - Review & Editing.

**Daniela Cristina Tartari:** Methodology, Formal analysis, Writing - Review & Editing.

**Sabrina de Souza:** Methodology, Formal analysis, Writing - Original Draft.

**Thiago Lopes Silva:** Methodology, Formal analysis, Writing - Original Draft.

**Andreia Tomazoni:** Methodology Formal analysis, Writing - Review & Editing.

**Patrícia Kuerten Rocha:** Conceptualization, Methodology, Formal analysis, Writing - Original Draft, Review & Editing, Supervision.

## Funding sources

This work was supported by the 10.13039/501100002322Coordination for the Improvement of Higher Education Personnel in Brazil (CAPES) – financial support through scholarships (COD FINANCE 001).

## Conflict of interest

The authors state that there is no conflict of interest.
