# Supplementary material for: Efficacy of disinfection procedures to reduce Acinetobacter baumanii blaOXA-23 contamination rate of needleless connectors: an in-vitro study
Source: Infect Prev Pract. 2023 Dec 1;6(1):100328. doi: 10.1016/j.infpip.2023.100328 (PMC10788527; doi:10.1016/j.infpip.2023.100328)
Supplement: Multimedia component 1 [file mmc1.docx]

Supplementary Table S1

Efficacy of disinfection procedures to reduce the load of *Acinetobacter baumanii bla*_OXA-23_ in two-way intermediate extender’s hub experimentally contaminated.

| Treatments | | *N* | cfu Median | % Reduction in bacterial load compared to NTC | Post-hoc analysis*:  Differences amongst treatments (p<0.05) |
| --- | --- | --- | --- | --- | --- |
| (1) | NTC | 7 | 342.0 | – | (2)(3)(4) |
| (2) | Sterile gauze with 70% ethanol | 7 | 176.0 | 48.54 | (1)(4) |
| (3) | Sterile gauze with Incidin® | 7 | 175.0 | 48.83 | (1)(4) |
| (4) | 70% IPA single-use cap | 8 | 43.5 | 87.28 | (1)(2)(3) |
| (5) | Neg C | 8 | 0 |  | – |

* Kruskal–Wallis test was performed with three degrees of freedom (DF) and *P*=0.000812.

Legend: NTC: Non-treated Control; N: total of experiments; CFU: Colony Forming Unit; IPA: Isopropyl Alcohol; Neg C: Negative Control.

Supplementary Table S2

Efficacy of disinfection procedures to reduce the load of *Acinetobacter baumanii bla*_OXA-23_ in needle-free valve experimentally contaminated

| Treatments | | *N* | cfu Median | % Reduction in bacterial load related to NTC | Post-hoc analysis**:  Differences amongst treatments (*P*<0.05) |
| --- | --- | --- | --- | --- | --- |
| (1) | NTC | 9 | 110 | – | (2)(3)(4) |
| (2) | Sterile gauze with 70% Ethanol | 9 | 3 | 97.27 | (1) |
| (3) | Sterile gauze with Incidin® | 9 | 4 | 96.36 | (1) |
| (4) | 70% IPA single-use cap | 9 | 6 | 94.54 | (1) |
| (5) | Neg C | 9 | – | – | – |

** Kruskal–Wallis test was performed with three degrees of freedom (DF) and *P*=0.000153.

Legend: NTC: Non-treated Control; N: total of experiments; CFU: Colony Forming Unit; IPA: Isopropyl Alcohol; Neg C: Negative Control.
